# Content analysis of promotional material for asthma-related products and therapies on Instagram

**DOI:** 10.1186/s13223-021-00528-3

**Published:** 2021-03-08

**Authors:** Brent Heineman, Marcella Jewell, Michael Moran, Kolbi Bradley, Kerry A. Spitzer, Peter K. Lindenauer

**Affiliations:** 1 Institute for Healthcare Delivery and Population Science, University of Massachusetts Medical School-Baystate, 3601 Main St, 3rd Floor, Springfield, MA 01107 USA; 2 University of Connecticut School of Medicine, Farmington, CT USA; 3 University of Massachusetts Medical School, Worcester, MA USA; 4 Department of Medicine, University of Massachusetts Medical School-Baystate, Springfield, MA USA; 5 Department of Population and Quantitative Health Sciences, University of Massachusetts Medical School, Worcester, MA USA

**Keywords:** Asthma, Social media, Clinical guidelines, Qualitative methods, Misinformation, Internet

## Abstract

**Background:**

Increasingly, social media is a source for information about health and disease self-management. We conducted a content analysis of promotional asthma-related posts on Instagram to understand whether promoted products and services are consistent with the recommendations found in the Global Initiative for Asthma (GINA) 2019 guidelines.

**Methods:**

We collected every Instagram post incorporating a common, asthma-related hashtag between September 29, 2019 and October 5, 2019. Of these 2936 collected posts, we analyzed a random sample of 266, of which, 211 met our inclusion criteria. Using an inductive, qualitative approach, we categorized the promotional posts and compared each post’s content with the recommendations contained in the 2019 GINA guidelines. Posts were categorized as “consistent with GINA” if the content was supported by the GINA guidelines. Posts that promoted content that was not recommended by or was unrelated to the guidelines were categorized as “not supported by GINA”.

**Results:**

Of 211 posts, 89 (42.2%) were promotional in nature. Of these, a total of 29 (32.6%) were categorized as being consistent with GINA guidelines. The majority of posts were not supported by the guidelines. Forty-one (46.1%) posts promoted content that was not recommended by the current guidelines. Nineteen (21.3%) posts promoted content that was unrelated to the guidelines. The majority of unsupported content promoted non-pharmacological therapies (n = 39, 65%) to manage asthma, such as black seed oil, salt-room therapy, or cupping.

**Conclusions:**

The majority of Instagram posts in our sample promoted products or services that were not supported by GINA guidelines. These findings suggest a need for providers to discuss online health information with patients and highlight an opportunity for providers and social media companies to promote evidence-based asthma treatments and self-management advice online.

## Introduction

Asthma affects more than 19 million adults in the United States and in 2016 resulted in greater than 1.2 million emergency department visits and 108,000 hospitalizations [1]. Effective self-management, including proper use of medications and avoidance of environmental triggers is a prerequisite for achieving optimal clinical outcomes [2].

Asthma is the most common chronic disease among youth and young adults. In 2018, over 1.7 million (8.1%) young adults ages 20–24 years reported having a current asthma diagnosis [1]. Young adults are more likely to be on social media and many turn to the Internet and social media for information about health and self-management [3]. Although a vast amount of health information is readily available at the click of a mouse, it is widely understood that the Internet is filled with a great deal of misinformation which may lead patients to make health decisions that run counter to treatment recommendations found in current guidelines [4]. With the increasing use of social media for health information, it is important to understand the nature of health information available on social media.

Instagram is a social media platform that allows users to share photos, videos, and text with followers. There are currently over 700,000 Instagram posts that incorporate asthma-related hashtags, many of which promote products and services. While Facebook remains the most popular social media [5] platform within the US, Instagram is the second most popular platform among young adults. In 2019, 75% of 18–24-year-olds used Instagram, compared to 76% who used Facebook [5]. Health-related content on Instagram and other social media platforms has been explored in previous studies. Many of these have examined the dissemination of information related to infectious diseases [6, 7] and mental health [8]. Yet to date, no studies have investigated the content of asthma-related posts on Instagram. Understanding the information to which patients with asthma are exposed on Instagram can help providers offer better guidance related to asthma self-management. Therefore, we conducted a qualitative content analysis of asthma-related posts on Instagram to determine whether they are consistent with the accepted, evidence-based 2019 GINA guidelines.

## Methods

Over a 1-week period between September 29, 2019 and October 5, 2019, we identified all Instagram posts containing one or more of the five most common asthma-related hashtags in the caption of the post: #asthma, #asthmasucks, #asthmaattack, #asthmatic, and #asthmaproblems. A 1-week period was chosen due to feasibility, as many posts are made daily containing these hashtags. Searches were conducted once a day during this 1-week period. Duplicates were avoided as all posts were saved in an Instagram collection, which prevents saving a post more than once. Of the 2936 posts screened, we randomly sampled every ninth post (n = 266). We excluded posts that were not in English, were concerned with asthma in animals, and one that appeared in our collection, but was outside of the study timeframe. A total of 211 posts met our inclusion criteria. All posts that met our inclusion criteria were uploaded to NVivo 12. Due to the original nature of this work, we used a conventional content analysis approach, meaning that codes were derived directly from the data [9]. In order to generate the initial codebook, four members of the team (BH, MJ, KS, and MM) independently reviewed the first day of posts and then came together to generate codes and definitions. Teams of two reviewed and coded each day and then the full team came back together to discuss any new codes and to ensure agreement on the classification of posts. Any instances of intercoder disagreement within the smaller teams were decided in on-going team coding meetings, the coding team membership changed over time to include a new member (KB).

**Fig. 1:**
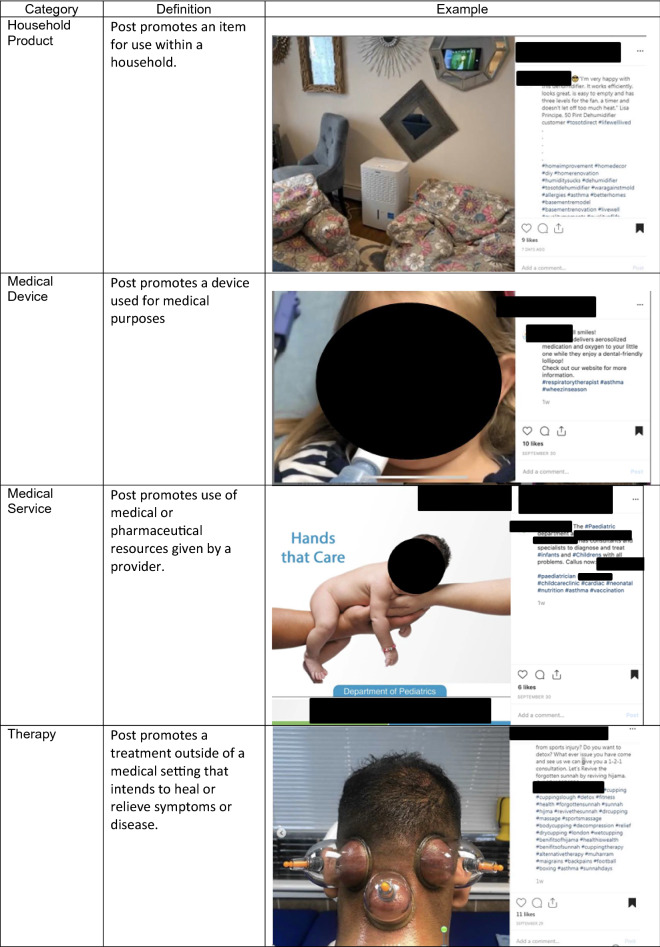
Examples of post type categorization

**Fig. 2:**
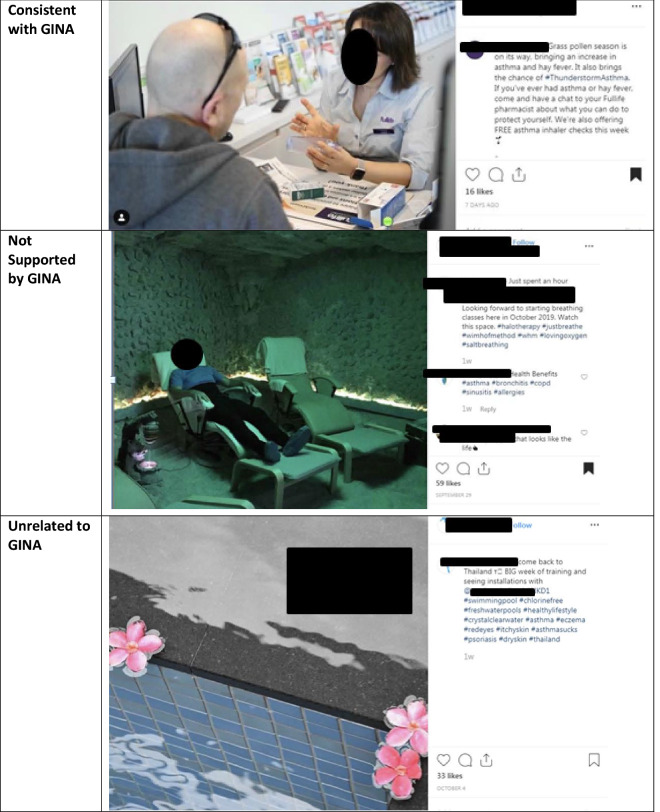
Examples of post categorizations based on GINA

This initial coding classified posts based on their purpose: “promotional” posts were defined as those that clearly sold, advertised, or recommended products, or services (n = 89), if a post was made by a for-profit entity it was considered promotional regardless of the content; “personal stories” were those that shared narratives from individuals living with asthma or caring for others with asthma (n = 88); “memes” were defined as funny or inspirational images, text, or videos that were copied and shared by Instagram users, sometimes alongside captions that shared personal stories or promotional content (n = 32); educational or advocacy posts were defined as those that shared asthma self-management strategies or advocated for greater awareness of asthma (n = 19); and posts that did not fit in these categories were classified as “other” (n = 7). Note that these classifications were not mutually exclusive and in categorizing posts the image or video was analyzed with the caption, hashtags, geotagged location, if available, and the poster’s identity. For example, if a commercial gym posted an image of weights with the hashtag “asthma” this would be coded as a promotional post, whereas an individual posting an image of themselves working out in the gym would be coded as a personal story. In some instances, personal stories were also coded as promotional, reflecting that the distinction between the personal and commercial is often blurry on social media. This research note analyzes the content of all posts that were classified as “promotional” (n = 89) in the context of the 2019 Global Initiative for Asthma (GINA) guidelines [10]. Members of the team (BH, MJ, KS, and MM) reviewed the first 20 promotional posts to further classify their content and specify what was being shared. After the promotional codes were generated, BH and MJ categorized each promotional post as relating to one of the following categories: household products; medical devices; medical services; pharmacologic therapies; non-pharmacologic therapies; or other (Fig. 1). Next, BH, MJ, and KB compared each post’s content to the recommendations presented in GINA and categorized posts as “consistent with GINA” if it promoted a product, therapy, or service that was recommended in the 2019 GINA guidelines and “not supported by GINA” if they promoted either a product or service that was not recommended by the guidelines, or if the post promoted something unrelated to the guidelines (Fig. 2). The full study team met regularly to discuss any discrepancies in the coding until consensus was reached. The University of Massachusetts-Baystate IRB committee approved this study as not human subjects research.

## Results

**Fig. 3:**
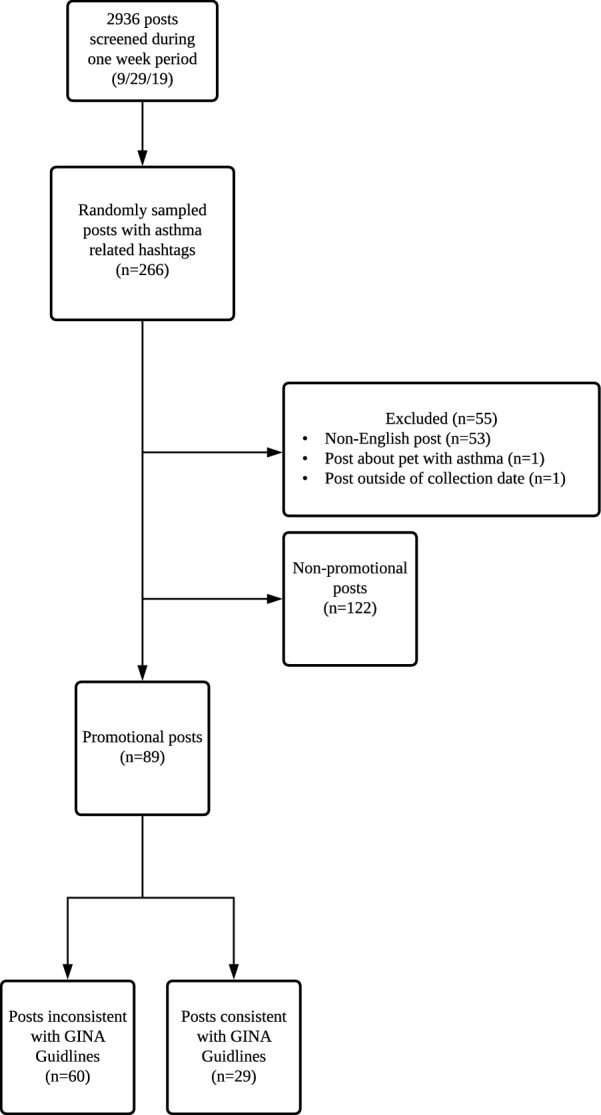
Collection and categorization of asthma-related Instagram posts

Our final sample included a total of 89 promotional posts, representing 42.2% of all English language posts with an asthma-related hashtag (Fig. 3).

A total of 29 (32.6%) promotional posts were consistent with the GINA guidelines. These posts included 9 promoting non-pharmacological therapies; 9 promoting medical services, 6 promoting a medical device, 1 promoting a pharmacological therapy, 2 promoting a household product and 2 promoting other content. An example of a medical device post was a photo of a portable compressor nebulizer that was described as “small, lightweight, fast and efficient nebulizer solution for active users who want to take their aerosol treatments anywhere and everywhere.” In another promotional example, a physician posted a patient testimonial about his successful asthma management plan.

The remaining 60 posts (67.4%) were not supported by current GINA guidelines in that they either were not recommended by the current guidelines (n = 41) or were unrelated to the current guidelines (n = 19). Of the posts that were not supported by the GINA guidelines, the majority promoted non-pharmacological therapies (n = 39). Ten posts promoted household products such as cleaning products, one promoted medical services, and 2 promoted pharmacological therapies, 9 posts promoted other content. Some examples of non-pharmacological therapies included “Hijima” or cupping sessions posted by an alternative and holistic health service user, halotherapy or salt room therapy posted by a yoga studio that owns a salt room, and 100% Pure Cold-Pressed, All Natural Nigella Sativa also described as black cumin seed oil posted by a personal account.

## Discussion

In this study of asthma-related posts on Instagram, we found that over 40% promoted products or services. Of concern, only one-third of the promoted content was supported by current GINA guidelines. Most of the “not supported by GINA” posts were non-pharmacological therapies including herbal medicine, acupuncture, and salt room therapy. There is scant evidence that alternative therapies improve asthma-related outcomes [11, 12]. These findings are significant given that approximately two-thirds of healthcare information seekers encounter health information on social media [13]. Moreover, a recent survey found that approximately one-third of Americans pursued health-related lifestyle changes due to information they read on social media [14].

While Americans continue to turn to social media for health information, the quality of information shared has come into question. Social media has been identified as a medium by which misinformation may easily spread [4]. This misinformation affects the dissemination of health information. Studies of diseases have shown that rumors and false information tend to have more shares on social media sites [15, 16]. Social media as a health information resource should, therefore, continue to be an area of interest for researchers and providers alike.

Providers play a critical role in disseminating health information and should employ health literacy practices to ensure that they are effectively communicating to patients. Based on the results of this analysis, we believe that providers should also inquire whether their patients turn to social media as a source for health information and should highlight the prevalence of ineffective and potentially harmful misinformation. During the COVID-19 pandemic, there has been much focus on the dissemination and spread of inaccurate information and conspiracy theories on social media [17–19]. Researchers and public health officials alike have warned of the negative effects of such false information on consumer health protective behaviors [20]. Due to this rampant misinformation, health organizations, such as the World Health Organization, are working with governments to reduce the spread of misinformation [21]. Facebook and other social media platforms have also increased efforts to combat misinformation and direct users to trusted sources [22, 23]. In tandem with this increased effort, social media companies should consider expanding the promotion of factual resources to other health conditions, including asthma.

This study has several limitations. Given Facebook’s and Instagram’s restrictions on automated collection of user information, we were restricted to manually gathering a small sample of public posts over a 1-week snapshot. This 1-week sample may not be representative of the posts made throughout the year, as the content of posts may shift depending on season or time of year. Additionally, our sample did not include private posts or paid advertisements, which may portray asthma-related material differently than the analyzed public posts. Furthermore, we categorized posts as either consistent with or inconsistent with the GINA guidelines, which precludes a more nuanced interpretation of the recommended good or service. Finally, posts that were categorized as not supported by GINA may be based on information published after the annual GINA guidelines and may be found within the upcoming versions. As a result, misinformation cannot be completely defined by inclusion within the 2019 GINA guidelines.

While this study focused on the content of promotional posts, the content of non-promotional posts is also deserving of study. Additionally, the content of comments on all posts is worth exploring further, as comments can be an additional source of misinformation. Overall, the importance of social media suggests these platforms as potentially effective means by which asthma advocacy organizations can connect directly with people living with asthma.

## Conclusion

In this qualitative content analysis of asthma-related promotional posts on Instagram, nearly 70% of the content recommended products and services that were not supported by or unrelated to the 2019 GINA guidelines. These findings shed additional light on the prevalence of misinformation and support a need to promote evidence-based resources online.

## Data Availability

The datasets used and/or analyzed during the current study are available from the corresponding author on reasonable request
